# Octa­kis(2-chloro­benz­yl)di-μ_2_-hydroxido-di-μ_3_-oxido-bis­(2-phenyl­acetato)tetra­tin(IV)

**DOI:** 10.1107/S1600536810012559

**Published:** 2010-04-17

**Authors:** Wei-Bing Peng, Guo-Qiang Li, Handong Yin, Xianhe Zhao

**Affiliations:** a Key Laboratory of Marine Drugs, Chinese Ministry of Education, Marine Drug and Food Institute, Ocean University of China, Qingdao 266003, People’s Republic of China; b College of Chemistry and Chemical Engineering, Liaocheng University, Shandong 252059, People’s Republic of China

## Abstract

The asymmetric unit of the title compound, [Sn_4_(C_7_H_6_Cl)_8_(C_8_H_7_O_2_)_2_O_2_(OH)_2_], comprises one-half of the centrosymmetric tin(IV) complex. μ_3_-Oxide and μ_2_-hydroxide bridges link the four five-coordinate Sn^IV^ atoms to generate three fused four-membered Sn—O—Sn—O rings in a ladder-like structure. The two endocyclic Sn atoms each bind to two μ_3_-oxide anions and a μ_2_-hydroxide ligand, together with two 2-chloro­benzyl groups. The exocyclic Sn atoms each carry a monodentate phenyl­acetate ligand, two 2-chloro­benzyl groups, and μ_3_-oxide and μ_2_-hydroxide ligands. Both types of Sn atoms adopt a distorted trigonal–bipyramidal coordination geometry. The mol­ecular conformation is stabilized by intra­molecular O—H⋯O inter­actions involving the μ_2_-hydroxide ligands and the C=O group of the phenyl­acetate ligand.

## Related literature

For the anti­fungal activity of organotin compounds, see: Ruzicka *et al.* (2002 ▶); Nath *et al.* (1999 ▶). For a related structure, see: Wu *et al.* (2009 ▶).
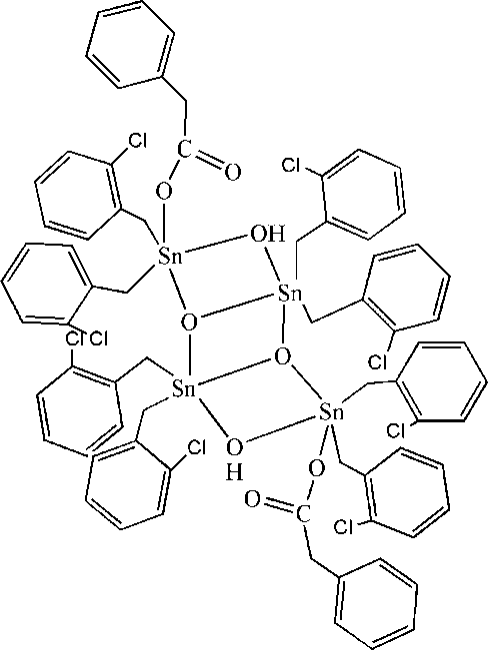


## Experimental

### 

#### Crystal data

[Sn_4_(C_7_H_6_Cl)_8_(C_8_H_7_O_2_)_2_O_2_(OH)_2_]
*M* *_r_* = 1815.59
Triclinic, 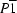

*a* = 10.7095 (14) Å
*b* = 11.4846 (16) Å
*c* = 15.2412 (18) Å
α = 98.311 (2)°
β = 90.982 (1)°
γ = 98.404 (2)°
*V* = 1833.6 (4) Å^3^
*Z* = 1
Mo *K*α radiation
μ = 1.69 mm^−1^
*T* = 298 K
0.49 × 0.48 × 0.40 mm

#### Data collection

Siemens SMART CCD area-detector diffractometer
Absorption correction: multi-scan (*SADABS*; Sheldrick, 1996 ▶) *T*_min_ = 0.491, *T*_max_ = 0.551
9422 measured reflections
6322 independent reflections
4437 reflections with *I* > 2σ(*I*)
*R*_int_ = 0.027

#### Refinement

*R*[*F* ^2^ > 2σ(*F* ^2^)] = 0.040
*wR*(*F* ^2^) = 0.121
*S* = 1.05
6322 reflections
415 parameters
H-atom parameters constrained
Δρ_max_ = 1.18 e Å^−3^
Δρ_min_ = −0.70 e Å^−3^

### 

Data collection: *SMART* (Siemens, 1996 ▶); cell refinement: *SAINT* (Siemens, 1996 ▶); data reduction: *SAINT*; program(s) used to solve structure: *SHELXS97* (Sheldrick, 2008 ▶); program(s) used to refine structure: *SHELXL97* (Sheldrick, 2008 ▶); molecular graphics: *SHELXTL* (Sheldrick, 2008 ▶); software used to prepare material for publication: *SHELXTL*.

## Supplementary Material

Crystal structure: contains datablocks I, global. DOI: 10.1107/S1600536810012559/sj2764sup1.cif

Structure factors: contains datablocks I. DOI: 10.1107/S1600536810012559/sj2764Isup2.hkl

Additional supplementary materials:  crystallographic information; 3D view; checkCIF report

## Figures and Tables

**Table 1:** Selected bond lengths (Å)

Sn1—O3	2.023 (4)
Sn1—O2	2.114 (4)
Sn1—C9	2.145 (6)
Sn1—C16	2.145 (7)
Sn1—O4	2.157 (3)
Sn2—O4	2.033 (4)
Sn2—O4^i^	2.089 (3)
Sn2—C30	2.146 (6)
Sn2—O3	2.163 (4)
Sn2—C23	2.165 (6)

Symmetry code: (i) 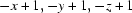.

**Table 2:** Hydrogen-bond geometry (Å, °)

*D*—H⋯*A*	*D*—H	H⋯*A*	*D*⋯*A*	*D*—H⋯*A*
O3—H3⋯O1	0.82	1.78	2.554 (7)	157

**Fig. 1.**
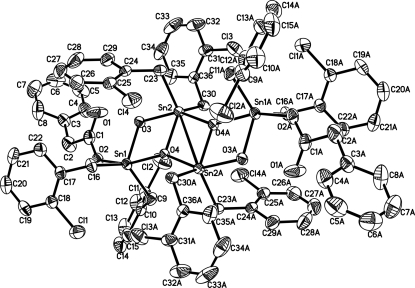
The molecular structure of (I), showing 50% probability displacement ellipsoids. H atoms have been omitted for clarity.

## supplementary crystallographic information

### Comment

Recently considerable attention has been paid to organotin(IV) derivatives, owing to their high in vitro antifungal activities against some medically important fungi (Ruzicka *et al.*, 2002; Nath *et al.*, 1999). As a continuation of our study of organotin compounds, we present here the synthesis and crystal structure of the title compound (I).

The title compound (Fig. 1, Table 1) is a centrosymmetric dimer and displays a ladder type structural motif. The ladder consists of four tin centers held together by two µ_3_-oxygen atoms. According to their different coordination environments, the four tin atoms can be divided into two types, viz. two endocyclic and two exocyclic. The endo- and exocyclic tin centers are linked by µ_2_-hydroxide anions and µ_3_-oxide anions. Each of the tin atoms is five-coordinate, adopting approximate trigonal bipyramidal coordination. The 2-phenylacetato ligands coordinate to the exocyclic tin atoms in a monodentate fashion, and the molecular conformation is stabilized by intramolecular O3—H3···O1 hydrogen bonds (Table 2). The crystal structure of a similiar compound has been reported recently (Wu *et al.*, 2009).

### Experimental

The reaction was carried out under a nitrogen atmosphere. 2-phenylacetic acid (2 mmol) and sodium ethoxide (2.2 mmol) were added to a stirred solution of benzene (30 ml) in a Schlenk flask and stirred for 0.5 h. Bis(2-chlorobenzyl)dichlorostannane (4 mmol) was then added to the reactor. After stirring for 10 h at 323 K, a white paste was obtained and filtered off. Colourless crystals suitable for X-ray analysis were obtained by slow evaporation of dichloromethane/methanol (1:1 v/v) solution over a period of six days (yield 86%. m.p. 438 K ).

### Refinement

H atoms were positioned geometrically, with C—H = 0.93, 0.97 and O—H = 0.82 Å for aromatic, methylene and hydroxyl H atoms, respectively, and constrained to ride on their parent atoms, with *U*_iso_(H) = 1.2 *U*_eq_(C) or 1.5 *U*_eq_(O) for hydroxyl groups

### Crystal data

**Table:**

[Sn_4_(C_7_H_6_Cl)_8_(C_8_H_7_O_2_)_2_O_2_(OH)_2_]	*Z* = 1
*M**_r_* = 1815.59	*F*(000) = 896
Triclinic, *P*1	*D*_x_ = 1.644 Mg m^−^^3^
Hall symbol: -P 1	Mo *K*α radiation, λ = 0.71073 Å
*a* = 10.7095 (14) Å	Cell parameters from 4098 reflections
*b* = 11.4846 (16) Å	θ = 2.3–27.0°
*c* = 15.2412 (18) Å	µ = 1.69 mm^−^^1^
α = 98.311 (2)°	*T* = 298 K
β = 90.982 (1)°	Block, colourless
γ = 98.404 (2)°	0.49 × 0.48 × 0.40 mm
*V* = 1833.6 (4) Å^3^	

### Data collection

**Table:**

Siemens SMART CCD area-detector diffractometer	6322 independent reflections
Radiation source: fine-focus sealed tube	4437 reflections with *I* > 2σ(*I*)
graphite	*R*_int_ = 0.027
φ and ω scans	θ_max_ = 25.0°, θ_min_ = 1.4°
Absorption correction: multi-scan (*SADABS*; Sheldrick, 1996)	*h* = −12→12
*T*_min_ = 0.491, *T*_max_ = 0.551	*k* = −7→13
9422 measured reflections	*l* = −18→17

### Refinement

**Table:**

Refinement on *F*^2^	Primary atom site location: structure-invariant direct methods
Least-squares matrix: full	Secondary atom site location: difference Fourier map
*R*[*F*^2^ > 2σ(*F*^2^)] = 0.040	Hydrogen site location: inferred from neighbouring sites
*wR*(*F*^2^) = 0.121	H-atom parameters constrained
*S* = 1.05	*w* = 1/[σ^2^(*F*_o_^2^) + (0.0507*P*)^2^ + 2.5952*P*] where *P* = (*F*_o_^2^ + 2*F*_c_^2^)/3
6322 reflections	(Δ/σ)_max_ = 0.001
415 parameters	Δρ_max_ = 1.18 e Å^−^^3^
0 restraints	Δρ_min_ = −0.70 e Å^−^^3^

### Fractional atomic coordinates and isotropic or equivalent isotropic displacement parameters (Å^2^)

**Table:**

	*x*	*y*	*z*	*U*_iso_*/*U*_eq_	
Sn2	0.37891 (4)	0.50151 (4)	0.43671 (3)	0.03405 (14)	
Sn1	0.52617 (4)	0.29250 (4)	0.32149 (3)	0.03909 (15)	
O1	0.2765 (5)	0.2430 (7)	0.1747 (4)	0.108 (3)	
Cl1	0.7318 (2)	0.0520 (2)	0.31794 (16)	0.0817 (7)	
Cl3	0.1945 (2)	0.7643 (2)	0.47210 (16)	0.0889 (7)	
O4	0.5297 (4)	0.4138 (3)	0.4439 (2)	0.0333 (9)	
O3	0.3714 (4)	0.3739 (4)	0.3168 (3)	0.0457 (11)	
H3	0.3230	0.3382	0.2763	0.069*	
C24	0.1584 (6)	0.3003 (6)	0.4525 (5)	0.0455 (16)	
C30	0.3829 (6)	0.6432 (6)	0.3587 (4)	0.0424 (15)	
H30A	0.4072	0.7196	0.3957	0.051*	
H30B	0.4431	0.6349	0.3122	0.051*	
O2	0.4756 (5)	0.2118 (4)	0.1896 (3)	0.0555 (13)	
C26	0.1527 (8)	0.0966 (8)	0.4741 (7)	0.077 (3)	
H26	0.1734	0.0419	0.5092	0.093*	
C20	0.4618 (10)	−0.1761 (7)	0.1799 (6)	0.077 (3)	
H20	0.4560	−0.2425	0.1363	0.092*	
C36	0.2531 (6)	0.6347 (6)	0.3195 (4)	0.0416 (15)	
C2	0.3728 (9)	0.1342 (8)	0.0521 (5)	0.078 (3)	
H2A	0.4492	0.1657	0.0250	0.094*	
H2B	0.3766	0.0512	0.0559	0.094*	
C32	0.0369 (8)	0.6721 (9)	0.3344 (7)	0.087 (3)	
H32	−0.0233	0.7094	0.3661	0.104*	
C23	0.1962 (6)	0.4311 (6)	0.4814 (5)	0.0479 (17)	
H23A	0.1975	0.4470	0.5457	0.057*	
H23B	0.1328	0.4728	0.4590	0.057*	
C31	0.1583 (7)	0.6850 (7)	0.3675 (5)	0.059 (2)	
C17	0.4781 (7)	0.0212 (6)	0.3103 (4)	0.0475 (17)	
C18	0.5837 (7)	−0.0225 (6)	0.2777 (5)	0.0513 (18)	
C1	0.3705 (8)	0.2012 (7)	0.1456 (5)	0.058 (2)	
C16	0.4855 (7)	0.1312 (6)	0.3779 (4)	0.0505 (18)	
H16A	0.5508	0.1298	0.4226	0.061*	
H16B	0.4058	0.1304	0.4073	0.061*	
C28	0.0522 (8)	0.1387 (11)	0.3465 (6)	0.089 (3)	
H28	0.0067	0.1113	0.2932	0.107*	
C25	0.1922 (7)	0.2164 (7)	0.4999 (5)	0.0556 (19)	
C22	0.3625 (8)	−0.0376 (7)	0.2728 (5)	0.063 (2)	
H22	0.2887	−0.0098	0.2917	0.075*	
C35	0.2176 (8)	0.5672 (7)	0.2378 (5)	0.064 (2)	
H35	0.2773	0.5319	0.2041	0.076*	
C29	0.0866 (7)	0.2605 (8)	0.3724 (5)	0.067 (2)	
H29	0.0628	0.3145	0.3378	0.081*	
C19	0.5779 (9)	−0.1213 (7)	0.2139 (5)	0.068 (2)	
H19	0.6511	−0.1498	0.1944	0.082*	
C21	0.3545 (9)	−0.1354 (7)	0.2086 (6)	0.074 (3)	
H21	0.2761	−0.1736	0.1849	0.089*	
Cl4	0.2865 (2)	0.2618 (2)	0.59644 (16)	0.0855 (7)	
Cl2	0.5677 (3)	0.5040 (3)	0.1524 (2)	0.1111 (10)	
C15	0.8327 (7)	0.2995 (7)	0.1657 (5)	0.064 (2)	
H15	0.8699	0.2630	0.2078	0.077*	
C10	0.7379 (7)	0.3672 (6)	0.1912 (4)	0.0533 (19)	
C9	0.7019 (6)	0.3859 (6)	0.2859 (4)	0.0531 (19)	
H9A	0.7004	0.4704	0.3031	0.064*	
H9B	0.7687	0.3647	0.3215	0.064*	
C3	0.2613 (8)	0.1411 (7)	−0.0071 (4)	0.058 (2)	
C11	0.6853 (8)	0.4162 (7)	0.1265 (5)	0.065 (2)	
C14	0.8730 (9)	0.2849 (8)	0.0798 (7)	0.083 (3)	
H14	0.9381	0.2412	0.0647	0.100*	
C4	0.2413 (9)	0.2473 (8)	−0.0274 (6)	0.078 (3)	
H4	0.2943	0.3160	−0.0024	0.093*	
C27	0.0837 (9)	0.0588 (9)	0.3973 (8)	0.090 (3)	
H27	0.0577	−0.0222	0.3794	0.108*	
C13	0.8155 (11)	0.3359 (9)	0.0172 (6)	0.094 (4)	
H13	0.8412	0.3257	−0.0410	0.112*	
C8	0.1816 (11)	0.0414 (8)	−0.0418 (6)	0.099 (3)	
H8	0.1937	−0.0325	−0.0279	0.119*	
C7	0.0792 (11)	0.0515 (12)	−0.0997 (7)	0.106 (4)	
H7	0.0231	−0.0159	−0.1231	0.127*	
C12	0.7206 (10)	0.4017 (9)	0.0394 (6)	0.087 (3)	
H12	0.6811	0.4357	−0.0032	0.104*	
C34	0.0942 (10)	0.5521 (9)	0.2061 (7)	0.087 (3)	
H34	0.0711	0.5061	0.1513	0.105*	
C33	0.0056 (10)	0.6040 (11)	0.2544 (9)	0.103 (4)	
H33	−0.0774	0.5925	0.2323	0.124*	
C5	0.1443 (11)	0.2550 (11)	−0.0843 (7)	0.098 (3)	
H5	0.1334	0.3291	−0.0985	0.118*	
C6	0.0637 (10)	0.1576 (14)	−0.1206 (6)	0.101 (4)	
H6	−0.0018	0.1643	−0.1595	0.121*	

### Atomic displacement parameters (Å^2^)

**Table:**

	*U*^11^	*U*^22^	*U*^33^	*U*^12^	*U*^13^	*U*^23^
Sn2	0.0364 (2)	0.0383 (3)	0.0277 (2)	0.00952 (19)	−0.00245 (17)	0.00236 (18)
Sn1	0.0458 (3)	0.0380 (3)	0.0327 (3)	0.0095 (2)	0.00195 (19)	−0.00089 (19)
O1	0.050 (4)	0.200 (8)	0.062 (4)	0.038 (4)	−0.011 (3)	−0.038 (4)
Cl1	0.0591 (13)	0.0889 (17)	0.0930 (17)	0.0115 (11)	0.0016 (12)	−0.0006 (13)
Cl3	0.0980 (18)	0.0925 (18)	0.0805 (16)	0.0418 (14)	0.0156 (13)	−0.0022 (13)
O4	0.040 (2)	0.035 (2)	0.025 (2)	0.0114 (18)	−0.0045 (17)	0.0011 (17)
O3	0.046 (3)	0.049 (3)	0.038 (2)	0.014 (2)	−0.009 (2)	−0.012 (2)
C24	0.035 (4)	0.048 (4)	0.052 (4)	0.002 (3)	0.007 (3)	0.009 (3)
C30	0.043 (4)	0.046 (4)	0.041 (4)	0.013 (3)	0.000 (3)	0.011 (3)
O2	0.072 (3)	0.050 (3)	0.042 (3)	0.010 (3)	−0.007 (3)	−0.005 (2)
C26	0.071 (6)	0.062 (6)	0.104 (8)	0.009 (5)	0.025 (5)	0.025 (5)
C20	0.111 (8)	0.047 (5)	0.067 (6)	0.004 (5)	−0.004 (6)	−0.001 (4)
C36	0.046 (4)	0.039 (4)	0.042 (4)	0.003 (3)	−0.006 (3)	0.016 (3)
C2	0.106 (7)	0.085 (6)	0.041 (5)	0.029 (5)	−0.004 (4)	−0.011 (4)
C32	0.054 (5)	0.109 (8)	0.112 (8)	0.016 (5)	−0.001 (5)	0.061 (7)
C23	0.041 (4)	0.056 (4)	0.048 (4)	0.009 (3)	0.008 (3)	0.009 (3)
C31	0.047 (4)	0.065 (5)	0.074 (5)	0.014 (4)	−0.002 (4)	0.031 (4)
C17	0.059 (4)	0.038 (4)	0.048 (4)	0.008 (3)	0.006 (3)	0.011 (3)
C18	0.057 (4)	0.049 (4)	0.048 (4)	0.009 (3)	0.002 (3)	0.005 (3)
C1	0.075 (6)	0.056 (5)	0.036 (4)	0.004 (4)	−0.009 (4)	−0.005 (3)
C16	0.068 (5)	0.038 (4)	0.048 (4)	0.011 (3)	0.015 (4)	0.008 (3)
C28	0.067 (6)	0.115 (9)	0.064 (6)	−0.026 (6)	0.010 (5)	−0.022 (6)
C25	0.046 (4)	0.056 (5)	0.065 (5)	0.002 (4)	0.019 (4)	0.011 (4)
C22	0.062 (5)	0.054 (5)	0.073 (5)	0.008 (4)	0.006 (4)	0.011 (4)
C35	0.082 (6)	0.059 (5)	0.049 (5)	−0.002 (4)	−0.021 (4)	0.018 (4)
C29	0.055 (5)	0.077 (6)	0.063 (5)	−0.001 (4)	0.007 (4)	−0.002 (4)
C19	0.089 (6)	0.055 (5)	0.063 (5)	0.027 (5)	0.016 (5)	−0.001 (4)
C21	0.084 (7)	0.050 (5)	0.080 (6)	−0.012 (5)	−0.013 (5)	0.006 (4)
Cl4	0.0702 (14)	0.1108 (19)	0.0823 (16)	0.0166 (13)	−0.0149 (12)	0.0361 (14)
Cl2	0.127 (2)	0.114 (2)	0.118 (2)	0.0578 (18)	0.0473 (18)	0.0594 (18)
C15	0.070 (5)	0.051 (5)	0.070 (5)	0.011 (4)	0.019 (4)	0.002 (4)
C10	0.063 (5)	0.051 (4)	0.039 (4)	−0.011 (4)	0.015 (3)	−0.002 (3)
C9	0.051 (4)	0.057 (5)	0.043 (4)	−0.005 (3)	0.007 (3)	−0.005 (3)
C3	0.079 (5)	0.064 (5)	0.032 (4)	0.019 (4)	−0.001 (4)	−0.003 (3)
C11	0.081 (6)	0.053 (5)	0.061 (5)	0.010 (4)	0.019 (4)	0.010 (4)
C14	0.098 (7)	0.066 (6)	0.082 (7)	0.008 (5)	0.041 (6)	−0.003 (5)
C4	0.099 (7)	0.070 (6)	0.064 (6)	0.020 (5)	0.000 (5)	0.002 (5)
C27	0.079 (7)	0.064 (6)	0.115 (9)	−0.015 (5)	0.043 (7)	−0.007 (6)
C13	0.144 (10)	0.074 (7)	0.054 (6)	−0.007 (7)	0.045 (6)	−0.002 (5)
C8	0.150 (10)	0.058 (6)	0.080 (7)	−0.002 (6)	−0.030 (7)	0.004 (5)
C7	0.114 (9)	0.111 (10)	0.077 (7)	−0.010 (7)	−0.028 (6)	−0.010 (7)
C12	0.119 (8)	0.088 (7)	0.053 (5)	0.009 (6)	0.018 (5)	0.015 (5)
C34	0.092 (7)	0.085 (7)	0.078 (7)	−0.024 (6)	−0.039 (6)	0.034 (5)
C33	0.058 (6)	0.125 (10)	0.130 (10)	−0.024 (6)	−0.039 (7)	0.077 (8)
C5	0.116 (9)	0.121 (10)	0.071 (7)	0.053 (7)	−0.007 (6)	0.024 (6)
C6	0.089 (8)	0.160 (12)	0.055 (6)	0.037 (8)	−0.008 (5)	0.006 (7)

### Geometric parameters (Å, °)

**Table:**

Sn1—O3	2.023 (4)	C18—C19	1.377 (10)
Sn1—O2	2.114 (4)	C16—H16A	0.9700
Sn1—C9	2.145 (6)	C16—H16B	0.9700
Sn1—C16	2.145 (7)	C28—C27	1.356 (14)
Sn1—O4	2.157 (3)	C28—C29	1.390 (12)
Sn2—O4	2.033 (4)	C28—H28	0.9300
Sn2—O4^i^	2.089 (3)	C25—Cl4	1.740 (8)
Sn2—C30	2.146 (6)	C22—C21	1.369 (11)
Sn2—O3	2.163 (4)	C22—H22	0.9300
Sn2—C23	2.165 (6)	C35—C34	1.377 (11)
Sn2—Sn2^i^	3.2130 (8)	C35—H35	0.9300
O1—C1	1.238 (9)	C29—H29	0.9300
Cl1—C18	1.745 (7)	C19—H19	0.9300
Cl3—C31	1.725 (8)	C21—H21	0.9300
O4—Sn2^i^	2.089 (3)	Cl2—C11	1.741 (8)
O3—H3	0.8200	C15—C14	1.379 (11)
C24—C25	1.370 (10)	C15—C10	1.395 (10)
C24—C29	1.412 (10)	C15—H15	0.9300
C24—C23	1.496 (9)	C10—C11	1.357 (11)
C30—C36	1.487 (8)	C10—C9	1.494 (9)
C30—H30A	0.9700	C9—H9A	0.9700
C30—H30B	0.9700	C9—H9B	0.9700
O2—C1	1.281 (8)	C3—C4	1.346 (11)
C26—C27	1.355 (13)	C3—C8	1.357 (11)
C26—C25	1.377 (11)	C11—C12	1.380 (11)
C26—H26	0.9300	C14—C13	1.371 (14)
C20—C21	1.359 (12)	C14—H14	0.9300
C20—C19	1.366 (11)	C4—C5	1.362 (12)
C20—H20	0.9300	C4—H4	0.9300
C36—C35	1.384 (9)	C27—H27	0.9300
C36—C31	1.405 (10)	C13—C12	1.371 (14)
C2—C3	1.503 (11)	C13—H13	0.9300
C2—C1	1.521 (9)	C8—C7	1.425 (14)
C2—H2A	0.9700	C8—H8	0.9300
C2—H2B	0.9700	C7—C6	1.335 (15)
C32—C33	1.358 (14)	C7—H7	0.9300
C32—C31	1.366 (11)	C12—H12	0.9300
C32—H32	0.9300	C34—C33	1.364 (15)
C23—H23A	0.9700	C34—H34	0.9300
C23—H23B	0.9700	C33—H33	0.9300
C17—C18	1.374 (9)	C5—C6	1.352 (14)
C17—C22	1.392 (10)	C5—H5	0.9300
C17—C16	1.502 (9)	C6—H6	0.9300
			
O4—Sn2—O4^i^	77.61 (16)	C17—C16—H16A	109.0
O4—Sn2—C30	120.9 (2)	Sn1—C16—H16A	109.0
O4^i^—Sn2—C30	103.2 (2)	C17—C16—H16B	109.0
O4—Sn2—O3	72.95 (15)	Sn1—C16—H16B	109.0
O4^i^—Sn2—O3	150.47 (16)	H16A—C16—H16B	107.8
C30—Sn2—O3	90.1 (2)	C27—C28—C29	121.5 (9)
O4—Sn2—C23	121.1 (2)	C27—C28—H28	119.3
O4^i^—Sn2—C23	101.5 (2)	C29—C28—H28	119.3
C30—Sn2—C23	116.6 (3)	C24—C25—C26	121.9 (8)
O3—Sn2—C23	95.6 (2)	C24—C25—Cl4	119.4 (6)
O4—Sn2—Sn2^i^	39.43 (10)	C26—C25—Cl4	118.7 (7)
O4^i^—Sn2—Sn2^i^	38.18 (11)	C21—C22—C17	121.7 (8)
C30—Sn2—Sn2^i^	118.26 (17)	C21—C22—H22	119.2
O3—Sn2—Sn2^i^	112.35 (11)	C17—C22—H22	119.2
C23—Sn2—Sn2^i^	117.14 (19)	C34—C35—C36	120.4 (9)
O3—Sn1—O2	86.93 (17)	C34—C35—H35	119.8
O3—Sn1—C9	117.5 (3)	C36—C35—H35	119.8
O2—Sn1—C9	93.6 (2)	C28—C29—C24	118.5 (9)
O3—Sn1—C16	111.2 (2)	C28—C29—H29	120.8
O2—Sn1—C16	95.2 (2)	C24—C29—H29	120.8
C9—Sn1—C16	130.8 (3)	C20—C19—C18	118.2 (8)
O3—Sn1—O4	73.29 (15)	C20—C19—H19	120.9
O2—Sn1—O4	159.50 (18)	C18—C19—H19	120.9
C9—Sn1—O4	90.9 (2)	C20—C21—C22	119.6 (8)
C16—Sn1—O4	96.9 (2)	C20—C21—H21	120.2
Sn2—O4—Sn2^i^	102.39 (16)	C22—C21—H21	120.2
Sn2—O4—Sn1	106.75 (16)	C14—C15—C10	122.0 (9)
Sn2^i^—O4—Sn1	150.5 (2)	C14—C15—H15	119.0
Sn1—O3—Sn2	106.89 (18)	C10—C15—H15	119.0
Sn1—O3—H3	109.5	C11—C10—C15	116.4 (7)
Sn2—O3—H3	142.1	C11—C10—C9	123.4 (7)
C25—C24—C29	118.0 (7)	C15—C10—C9	120.2 (8)
C25—C24—C23	122.7 (6)	C10—C9—Sn1	118.6 (4)
C29—C24—C23	119.2 (7)	C10—C9—H9A	107.7
C36—C30—Sn2	106.7 (4)	Sn1—C9—H9A	107.7
C36—C30—H30A	110.4	C10—C9—H9B	107.7
Sn2—C30—H30A	110.4	Sn1—C9—H9B	107.7
C36—C30—H30B	110.4	H9A—C9—H9B	107.1
Sn2—C30—H30B	110.4	C4—C3—C8	119.5 (9)
H30A—C30—H30B	108.6	C4—C3—C2	119.6 (8)
C1—O2—Sn1	129.9 (5)	C8—C3—C2	120.9 (8)
C27—C26—C25	119.8 (9)	C10—C11—C12	123.5 (8)
C27—C26—H26	120.1	C10—C11—Cl2	119.6 (6)
C25—C26—H26	120.1	C12—C11—Cl2	116.8 (8)
C21—C20—C19	121.2 (8)	C13—C14—C15	118.9 (9)
C21—C20—H20	119.4	C13—C14—H14	120.5
C19—C20—H20	119.4	C15—C14—H14	120.5
C35—C36—C31	117.1 (7)	C3—C4—C5	120.6 (9)
C35—C36—C30	121.3 (7)	C3—C4—H4	119.7
C31—C36—C30	121.4 (6)	C5—C4—H4	119.7
C3—C2—C1	114.5 (7)	C26—C27—C28	120.2 (9)
C3—C2—H2A	108.6	C26—C27—H27	119.9
C1—C2—H2A	108.6	C28—C27—H27	119.9
C3—C2—H2B	108.6	C12—C13—C14	120.8 (8)
C1—C2—H2B	108.6	C12—C13—H13	119.6
H2A—C2—H2B	107.6	C14—C13—H13	119.6
C33—C32—C31	118.9 (10)	C3—C8—C7	119.1 (10)
C33—C32—H32	120.5	C3—C8—H8	120.5
C31—C32—H32	120.5	C7—C8—H8	120.5
C24—C23—Sn2	114.0 (4)	C6—C7—C8	120.2 (10)
C24—C23—H23A	108.8	C6—C7—H7	119.9
Sn2—C23—H23A	108.8	C8—C7—H7	119.9
C24—C23—H23B	108.8	C13—C12—C11	118.4 (10)
Sn2—C23—H23B	108.8	C13—C12—H12	120.8
H23A—C23—H23B	107.7	C11—C12—H12	120.8
C32—C31—C36	122.1 (8)	C33—C34—C35	120.6 (9)
C32—C31—Cl3	118.4 (7)	C33—C34—H34	119.7
C36—C31—Cl3	119.5 (5)	C35—C34—H34	119.7
C18—C17—C22	116.3 (6)	C32—C33—C34	120.9 (9)
C18—C17—C16	122.6 (6)	C32—C33—H33	119.5
C22—C17—C16	121.0 (7)	C34—C33—H33	119.5
C17—C18—C19	123.1 (7)	C6—C5—C4	121.5 (11)
C17—C18—Cl1	118.5 (5)	C6—C5—H5	119.2
C19—C18—Cl1	118.5 (6)	C4—C5—H5	119.2
O1—C1—O2	124.1 (6)	C7—C6—C5	119.1 (11)
O1—C1—C2	122.6 (7)	C7—C6—H6	120.5
O2—C1—C2	113.2 (7)	C5—C6—H6	120.5
C17—C16—Sn1	113.0 (5)		

Symmetry codes: (i) −*x*+1, −*y*+1, −*z*+1.

### Hydrogen-bond geometry (Å, °)

**Table:**

*D*—H···*A*	*D*—H	H···*A*	*D*···*A*	*D*—H···*A*
O3—H3···O1	0.82	1.78	2.554 (7)	157
